# Protective effect of *Allium hookeri* water extract and its main compound, Cycloalliin, on foam cell formation in THP-1-derived macrophages

**DOI:** 10.29219/fnr.v69.10763

**Published:** 2025-05-27

**Authors:** Ha-Rin Moon, Jung-Mi Yun

**Affiliations:** 1 Department of Food and Nutrition, Chonnam National University, Yongbong-ro, Buk-gu, Gwangju, South Korea; 2 Technology Innovation Research Division, World Institute of Kimchi, 86 Kimchi-ro, Nam-gu, Gwangju, South Korea

**Keywords:** atherosclerosis, Allium hookeri, cycloalliin, foam cells, oxidized low-density lipoprotein, THP-1

## Abstract

**Background:**

Low-density lipoproteins are oxidized and modified by macrophages. This process leads to the formation of macrophage-derived cholesterol-rich foam cells, which are a hallmark of early atherosclerosis. The accumulation of these form cells plays a crucial role in atherosclerosis progression. *Allium hookeri* (*A. hookeri*), a medicinal herb commonly used in Southeast Asia, is known for its various bioactive effects, including antioxidant, antibacterial, and antidiabetic properties. However, the repressive effect of *A. hookeri* extract on foam cell formation in THP-1 macrophages remains unclear.

**Objective:**

This study aims to explore the effect of *A. hookeri* hot water extract (AHWE) and its primary compound, cycloalliin, on foam cell formation. This investigation involves a combined treatment of oxidized low-density lipoprotein and lipopolysaccharide to stimulate the development of atherosclerosis *in vitro.* Additionally, the regulatory mechanisms underlying this process were elucidated.

**Design:**

THP-1 cells were differentiated by phorbol 12-myristate 13-acetate (PMA) (1 μM) for 48 h. Subsequently, they were treated with either AHWE or cycloalliin for 48 h. THP-1 macrophages were treated with combined ox-LDL (20 μg/mL) and LPS (500 ng/mL) for 24 h. Cell viability was assessed using MTT assays, while lipid accumulation was visualized through Oil Red O staining. The levels of corresponding proteins and mRNA were quantified using western blotting and quantitative polymerase chain reactions.

**Results:**

THP-1 cells were differentiated with PMA (1 μM) for 48 h and then treated with or without AHWE and cycloalliin for 48 h. Subsequently, THP-1 macrophages were treated with combined ox-LDL (20 μg/mL) and LPS (500 ng/mL) for 24 h before harvesting. Ox-LDL and LPS treatment for 24 h enhanced the lipid accumulation in foam cells compared to those in untreated cells using Oil red O staining. Conversely, AHWE and cycloalliin treatment inhibited lipid accumulation in foam cells. These treatments significantly upregulated cholesterol efflux-related genes, including ATP binding cassette subfamily A member 1 (ABCA1), liver-X-receptor ɑ (LXRɑ), and peroxisome proliferator-activated receptor gamma (PPARγ) expression. Additionally, AHWE and cycloalliin decreased lipid accumulation-related genes, including lectin-like oxidized low-density lipoprotein receptor-1 (LOX-1), cluster of differentiation 36 (CD36), and scavenger receptor A1 (SR-A1) expression. Furthermore, the combined treatment of ox-LDL and LPS increased the activation and expression of nuclear factor-κB (NF-κB), cyclooxygenase-2 (COX-2), and pro-inflammatory cytokines (tumor necrosis factor-α [TNF-α] and IL-6) compared with those in untreated cells. However, AHWE and cycloalliin suppressed the expression of NF-κB, COX-2, TNF-α, and IL-6.

**Conclusions:**

AHWE and cycloalliin potentially play a crucial role in suppressing and protecting against early-stage foam cell formation by modulating lipid accumulation and cholesterol efflux. AHWE and cycloalliin have the potential to be effective agents for preventing atherosclerosis.

## Popular scientific summary

- *Allium hookeri* water extract (AHWE) and cycloalliin improve cholesterol efflux in ox-LDL-loaded macrophages by upregulating ABCA1/LXRα/PPAR expression and simultaneously suppressing lipid uptake by increasing levels of SR-A1, CD36, and LOX-1 in THP-1 macrophages.
- AHWE and cycloalliin jointly inhibit proinflammatory events by modulating NF-κB expression and inhibiting cytokine release in co-treated ox-LDL and LPS-induced foam cells.
- AHWE and cycloalliin have the potential to be effective agents for preventing atherosclerosis.

Cardiovascular disease is a leading cause of death in several countries (1). Atherosclerosis, a condition characterized by persistent inflammation within the arterial walls and buildup of excessive cholesterol, serves as a significant risk factor for cardiovascular disease and progresses over time (2).

Monocytes play a crucial role in the development of early atherosclerotic lesions and disease progression (3, 4). When endothelial cells suffer damage from risk factors such as hyperlipidemia, monocytes infiltrate the inner wall of blood vessels and differentiate into macrophages (5). Low-density lipoprotein (LDL) within the intimal layer is oxidized by macrophages, resulting in its conversion to oxidized low-density lipoprotein (ox-LDL) (6). This ox-LDL consequently accelerates the formation of foam cells within damaged endothelial cells through the phagocytic activity of macrophages. These foam cells then accumulate within the inner wall of blood vessels (7). The formation of foam cells within the endothelium marks a crucial stage in the initiation and progression of early atherosclerosis (8).

An imbalance between lipid accumulation and cholesterol efflux is associated with foam cell development (9). Therefore, restoring the balance between lipid accumulation and cholesterol efflux may be an important strategy for preventing early atherosclerosis. Macrophages internalize ox-LDL molecules through the scavenger receptor (SR) pathway, leading to their transformation into lipid-laden foam cells (10). In cellular and animal studies, elevated levels of SR expression increase lipid accumulation and expedite the formation of atherosclerotic lesions (11). Various SRs, including scavenger receptor class A1 (SR-A1), cluster of differentiation 36 (CD36), and lectin-like oxidized low-density lipoprotein receptor-1 (LOX-1), bind to ox-LDL, facilitating the uptake of LDL and promoting the translation of macrophages into foam cells (12, 13). Additionally, SRs expressed on macrophage surfaces upregulate inflammatory cytokines, further inducing LDL accumulation by macrophages (14).

Cholesterol efflux reduces the accretion of cholesterol esters in macrophages, thereby inhibiting their transformation into foam cells (15). Cellular and clinical studies have highlighted the importance of inducing cholesterol efflux in preventing atherosclerosis (16–18). In macrophages, cholesterol efflux to extracellular receptors is facilitated by active transport through ATP-binding cassette transporter A1 (ABCA1) and ATP-binding cassette transporter G1 (ABCG1)-containing transporters (19). The transcription of ABCA1 and ABCG1, crucial for cholesterol efflux into the extracellular space, is induced by peroxisome proliferator-activated receptor-γ (PPARγ) and liver X receptor-α (LXRα) (20). This PPARγ/LXRα/ABCA1 pathway significantly influences atherosclerotic plaque formation by stimulating cholesterol efflux from macrophages (21).

The inflammatory response is pivotal in atherosclerosis onset and progression, as it stimulates macrophage lipid uptake and foam cell formation, contributing to endothelial dysfunction (22). Damage to the endothelium leads to increased CD36 expression, which in turn elevates the secretion of inflammatory cytokines such as TNF-α and IL-1β, further promoting foam cell formation (23, 24). Additionally, foam cells release reactive oxygen species that trigger LDL oxidation, exacerbating the secretion of inflammatory cytokines and promoting atherosclerosis progression (22). Ox-LDL prompts nuclear factor-κB (NF-κB) signaling, enhancing cytokine production and fostering atherosclerosis development (25). NF-κB, a crucial inflammation regulator, serves as a pivotal transcription factor in plaque formation and atherosclerosis progression. Activation of NF-κB accelerates atherosclerotic plaque formation by increasing the expression of pro-inflammatory cytokines, including interleukin-12, tumor necrosis factor-α (TNF-α), and monocyte chemoattractant protein-1 in macrophages (26–28). Several recent studies have underscored the pivotal role of Sirtuin-1 (SIRT1) as a regulator in the formation and progression of atherosclerosis. SIRT1, present in macrophages and endothelial cells, suppresses NF-κB transcriptional activity and curbs inflammatory responses by regulating pro-inflammatory factors, including IL-6 and TNF-ɑ. A recent study observed reduced levels of NF-κB and inflammatory cytokines in SIRT1-overexpressing mice fed a high-fat diet, leading to reduced hepatic lipid accumulation (29). Therefore, promoting cholesterol efflux from foam cells and suppressing lipid accretion and inflammation may serve as strategies to prevent atherosclerosis progression.

Anti-atherosclerosis medications effectively lower cholesterol levels; however, they are associated with side effects such as cognitive impairment, liver toxicity, and diabetes (30, 31). Recent research has actively focused on exploring natural ingredients devoid of such adverse effects. *Allium hookeri (A. hookeri),* indigenous to Southeast Asia, has long been utilized both as a culinary ingredient and in traditional medicine for purposes such as fatigue recovery and immunity enhancement (32, 33). *A. hookeri* contains natural compounds such as saponin and unique amino acids including S-Allyl-l-cysteinsulfoxide and cycloalliin (34–36). Recently, several biological activities, such as antibacterial, anti-dementia, and lipid peroxidation inhibition, have been reported in the roots of *A. hookeri* (37–39). Cycloalliin, which is abundantly contained in *A. hookeri*, is one of the main active ingredients (40). Cycloalliin, is contained at 5.44% based on the freeze-dried sample of *A. hookeri* root water extract and is stable to heat and moisture (41). Cycloalliin is a sulfur-containing cyclic imino compound that is reported to have antioxidant, anti-inflammatory, and anticancer effects (42, 43). However, studies on the inhibitory effects of *A. hookeri* root extract and cycloalliin on foam cell formation in macrophages are limited. Therefore, this study aimed to investigate the mechanism by which *A. hookeri* root extract and its major component, cycloalliin, affects cholesterol efflux, lipid accumulation, and inflammation-related gene expression on foam cell formation in THP-1-derived macrophages.

## Materials and methods

### Materials

Human THP-1 cells were procured from the Korean Cell Line Bank (Seoul, South Korea). *A. hookeri* root dry powder used in this study was purchased from an online open market (Gmarket, Seoul, Korea). Cycloalliin was purchased from Fuji Film Wako Chemicals (Osaka, Japan). LPS, phorbol 12-myristate 13-acetate (PMA), and thiazolyl blue tetrazolium bromide (MTT) were purchased from Sigma-Aldrich (St. Louis, MO, USA). The BCA protein assay kit and ox-LDL were obtained from Thermo Fisher Scientific (Waltham, MA, USA). The SR-A1, CD36, ABCA1 were purchased from Abcam (Cambridge, UK). LOX-1, LXR-α, PPARγ, TNF-α, COX-2, and NF-κB were obtained from Santa Cruz Biotechnology (Santa Cruz, CA, USA). SIRT1 and secondary antibodies were purchased from Cell Signaling Technology (Beverly, MA, USA). Quantitative polymerase chain reaction (qPCR) primers (ABCA1, PPAR-γ, LXR-α, LOX-1, CD36, TNF-α, COX-2, and β-actin) were sourced from Bioneer (Daejeon, South Korea). Unless specifically mentioned, extraction solvents and all other chemicals were obtained from Sigma-Aldrich or Biosesang (Sungnam, Gyeonggi-do, South Korea).

### *A. hookeri* root extract preparation

The *A. hookeri* root hot water extract (AHWE) was prepared by adding 10 times the amount of water per gram of powders, stirring twice in reflux, cooling at 95°C for 4 h, and then filtering the mixture. The resulting extract was concentrated under reduced pressure using a rotary vacuum evaporator (EYELA N-1000, Tokyo, Japan) and subsequently dried to determine the solid content. The yields of AHWE were calculated to be 34.25%. Store at -80°C for use in further experimental analysis.

### THP-1 cell culture and PMA-induced differentiation

Human THP-1 cells were cultured in RPMI 1640 medium (Welgene, Daegu, South Korea) supplemented with 10% fetal bovine serum and 1% antibiotics (Welgene). The culture was maintained in an atmosphere of 5% CO_2_ at 37°C. To induce differentiation into macrophages, THP-1 cells were treated with PMA (1 μM) for 48 h. Following differentiation, THP-1 cells were cultured in the presence or absence of various concentrations of AHWE (31–125 μg/mL) and cycloallin (2.5–10 μM) for 48 h. Subsequently, the cells were treated with ox-LDL (20 μg/mL) and LPS (500 ng/mL) for 24 h before harvest. Upon completion of the treatment, the culture medium was collected for cytokine secretion measurement, and the cells were washed twice with phosphate-buffered saline (PBS; Biosesang) before being harvested.

### Cell viability measurement

The cytotoxic effects of AHWE and cycloalliin on PMA-activated THP-1 macrophages were assessed using the MTT assay. The cells were seeded at 1×10^6^ cells/well in 24-well plates and treated with AHWE and cycloalliin for 48 h. Subsequently, the cells were co-treated with ox-LDL (20 μg/mL) and LPS (500 ng/mL) for 24 h before the MTT assay. Following treatment, MTT solution (100 μL; 1 mg/mL) was added and incubated for an additional 2 h. The precipitated formazan was solubilized in 1mg/mL of 100% dimethyl sulfoxide (DMSO). Finally, the absorbance at 570 nm was measured using a plate reader (EZRead 400 microplate reader, Biochrom, Cambridge, UK).

### Oil red O staining

Oil Red O staining was performed to assess lipid accumulation in macrophage-derived foam cells. Cells were examined for lipid inclusion through Oil Red O staining. Briefly, the cells were fixed with 4% paraformaldehyde (PFA) for 30 min at 4°C, followed by treatment with Oil Red O solution (Sigma-Aldrich) for 30 min. Images were captured using a Leica microscope, and data acquisition was facilitated by the Leica Application Suite X software (Leica Microsystems, Wetzlar, Germany). A 400× objective was utilized for image acquisition. The staining degree was quantified by measuring absorbance at 520 nm using an EZRead 400 microplate reader.

### Enzyme-linked immunosorbent assay

Cell-free supernatants were collected, and cytokine levels were measured using IL-6 and TNF-α ELISA Kits (Raybiotech, Norcross, GA, USA) to evaluate the influence of AHWE and cycloalliin on cytokine production in PMA-activated THP-1 macrophages. In detail, 100 μL of each sample was added to the pre-coated wells and incubated at room temperature for 2 h. After washing, biotinylated detection antibodies were added and then incubated with the substrate. Cytokine production was quantified by measuring absorbance at 450 nm using an EZRead 400 microplate reader.

### Immunoblotting analysis

Immunoblotting analysis was conducted to determine the expression levels of proteins associated with inflammation, lipid accumulation, and cholesterol efflux. Whole-cell lysates were prepared using RIPA buffer (Biosesang, Sungnam, Korea) supplemented with Halt™ protease and phosphatase inhibitor cocktail (Thermo Fisher Scientific, Waltham, MA, USA). For nuclear lysates, a nuclear extraction buffer containing (20 mM HEPES, 0.4 mM NaCl, 1 mM EDTA, 1 mM EGTA, 1 mM dithiothreitol, and 1 mM PMSF) and 10% NP-40 was used. Lysate protein concentrations were determined using a BCA protein assay (Pierce, IL, USA) following the instruction of the manufacturer. For SDS-PAGE, proteins (20 μg) were separated and transferred into a nitrocellulose membrane (Invitrogen, Waltham, MA, USA). The membrane was then blocked for 2 h in a buffer containing (10 mM Tris-HCl [pH 7.5], 150 mM NaCl, 0.1% Tween 20, and 5% nonfat dry milk), followed by incubation with primary antibodies for 2 h. Subsequently, the membrane was washed and incubated with a diluted conjugated secondary antibody for 2 h. After application of the Western blotting luminol reagent (Santa Cruz Biotechnology, Dallas, TX, USA), the results were analyzed using the ChemiDoc XRS+ Imaging System (BioRad, Hercules, CA, USA). Protein expression intensity was normalized to *β*-actin. Subsequently, it was quantified using ImageJ (a free online image analysis software). The following primary antibodies were used: SR-A1 (abcam Cat#ab151707, 1:1000 dilution), CD36 (abcam Cat#ab133625, 1:1000 dilution), LOX-1 (Santa Cruz Cat#sc-66155, 1:500 dilution), ABCA1 (abcam Cat# ab18180, 1:1000 dilution), LXR-α (Santa Cruz Cat#sc-377260, 1:500 dilution), PPARγ (Santa Cruz Cat#sc-7273, 1:1000 dilution), TNF-α (Santa Cruz Cat#sc-133192, 1:1000 dilution), COX-2 (Santa Cruz Cat#sc-376861, 1:500 dilution), NF-κB (Santa Cruz Cat#sc-8008, 1:1000 dilution), and SIRT1 (Cell signaling technology Cat #2310S, 1:1000 dilution). The secondary antibody used was Anti-rabbit IgG, HRP-linked Antibody (Cell Signaling technology Cat# 7074P2, 1:2000 dilution), Anti-mouse IgG, HRP-linked Antibody (Cell Signaling technology Cat# 7076P2, 1:2000 dilution).

### Quantitative polymerase chain reaction analysis

qPCR was conducted to assess mRNA levels of genes associated with inflammation, cholesterol efflux, and lipid accumulation. Total RNA was isolated using a Trizol reagent following the instruction of the manufacturer (Thermo Fisher Scientific, Waltham, MA, USA). Total RNA concentration and purity were assessed by measuring absorbance at 260 and 280 nm using a NanoDrop 2000 spectrophotometer (Thermo Fisher Scientific, Waltham, MA, USA). First-strand cDNA synthesis was performed using an Omniscript RT kit (QIAGEN, Hilden, Germany) with 1 μg of total RNA. SYBR green-based quantitative PCR was performed using a CFX96 Touch Real-Time PCR Detection System (Bio-rad, CA, USA). All reactions were performed in triplicate. Statistical significance was determined by comparing the β-actin-normalized 2^-∆∆CT^ values. The primer sequences were as follows: human *ABCA1*, forward 5’-GTCCTCTTTCCCGCATTATCTGG -3’ and reverse 5’- AGTTCCTGGAAGGTCTTGTTCAC-3’; *PPAR-γ*, forward 5’- CACAAGAACAGATCCAGTGGTTGCAG-3’ and reverse 5’- AATAATAAGGTGGAGATGCAGGCTCC-3’; *LXR-α*, forward 5’- ACACCTACATGCGTCGCAAG-3’ and reverse 5’- GACGAGCTTCTCGATCATGCC-3’; *LOX-1*, forward 5’- GGGCTCATTTAACTGGGAAA-3’ and reverse 5’-GAAATTGCTTGCTGGATGAA-3’; *CD36*, forward 5’-GGGAAAGTCACTGCGACATG-3’ and reverse 5’-TGCAATACCTGGCTTTTCTCA-3’; *TNF-α*, forward 5’-CAATGTAGGAGCTGCCTTGG-3’ and reverse 5’-CAGAGGCTCAGCAATGAGTG-3’; *COX-2*, forward 5’- AGATCATCTCTGCCTGAGTATCTT-3’ and reverse 5’- TTCAAATGAGATTGTGGGAAAATTGCT-3’; *β-actin*, forward 5’- CACCCCGTGCTGCTGAC-3’ and reverse 5’- CCAGAGGCGTACAGGGATAG-3’.

### Immunofluorescence staining

Immunofluorescence staining was performed to observe the nuclear translocation of transcription factor NF-κB in the cell nucleus. After AHWE and cycloalliin treatment, cells were washed twice in PBS, fixed with 4% PFA for 30 min at 4°C, and stained overnight with the NF-κB antibody (1:100 dilution, Santa Cruz Biotechnology, Dallas, TX, USA). After air drying, the slides were incubated with a secondary antibody (1:2000 dilution, Invitrogen, USA) for 60 min. Following this, DAPI (100 ng/mL, Beyotime, Shanghai, China) was used to stain the nuclei at 37°C, and the samples were washed thrice with PBS. The slides were then washed twice in PBS, air-dried, treated with a mounting medium, and examined at 400× magnification under a fluorescence microscope. The Leica Application Suite X software was utilized to collect images.

### Statistical analysis

All experiments were conducted independently a minimum of three times, and the results were presented as mean ± standard deviation. Significant differences among groups were determined through one-way ANOVA, followed by the Duncan multiple range test using SPSS version 25.0 (SPSS Institute, Chicago, IL, USA). The specific significance values are provided in the figure legend, and statistical significance was defined as *P* < 0.05.

## Results

### AHWE and cycloalliin inhibit inflammation in LPS-induced THP-1 cells

**Fig. 1:**
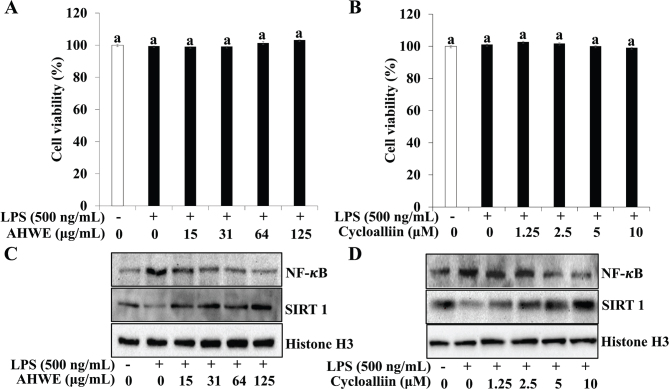
Effect of LPS on cell viability of THP-1 cells and upregulated expression of inflammatory factor. (A–B) THP-1 monocytes were exposed to 1 μM of PMA for 48 h and then pretreated with various concentrations of AHWE and cycloalliin before being stimulated with or without 500 ng/mL LPS for 24 h. Cell viability was assessed using the MTT assay. Experiments were performed in triplicate, and the results are presented as the mean ± SD. Significant differences (*P* < 0.05) were identified using Duncan’s multiple range test, with distinct letters indicating significance. The protein expression levels of NF-κB and SIRT1 were analyzed using (C–D) immunoblotting. (C) NF-κB and (D) SIRT1 levels. LPS, lipopolysaccharides; AHWE, *Allium hookeri* hot water extract; NF-κB, nuclear factor-κB; SIRT1, sirtuin 1; PMA, phorbol 12-myristate 13-acetate; MTT, 3-(4,5-Dimethyl-2-thiazolyl)-2,5-diphenyl-2H-tetrazolium Bromide; SD, Standard deviation.

The cytotoxicity of AHWE and cycloalliin was assessed in an inflammatory environment induced by LPS. Studies with mouse skin cells and human lung cancer cells, cycloalliin was used at concentrations ranging from 0.034 to 100 μM (44, 45). In our study, the MTT experiment showed no toxicity to cells at concentrations less than 10 μM. No cytotoxic effects were observed for either AHWE or cycloalliin in the LPS-treated and untreated groups (Fig. 1A–B). Consequently, the subsequent experiments utilized the non-toxic concentration range of AHWE (31–125 μg/mL) and cycloalliin (2.5–10 μM). To investigate whether AHWE and cycloalliin could inhibit LPS-induced NF-κB and SIRT1 expression in the nucleus, western blotting was performed. Figure 1C–D shows that NF-κB expression increased while SIRT1 expression decreased in the LPS-induced inflammatory environment. However, treatment with AHWE and cycloalliin resulted in a decrease in NF-κB expression and an increase in SIRT1 expression.

### AHWE and cycloalliin effects on foam cell formation

**Fig. 2:**
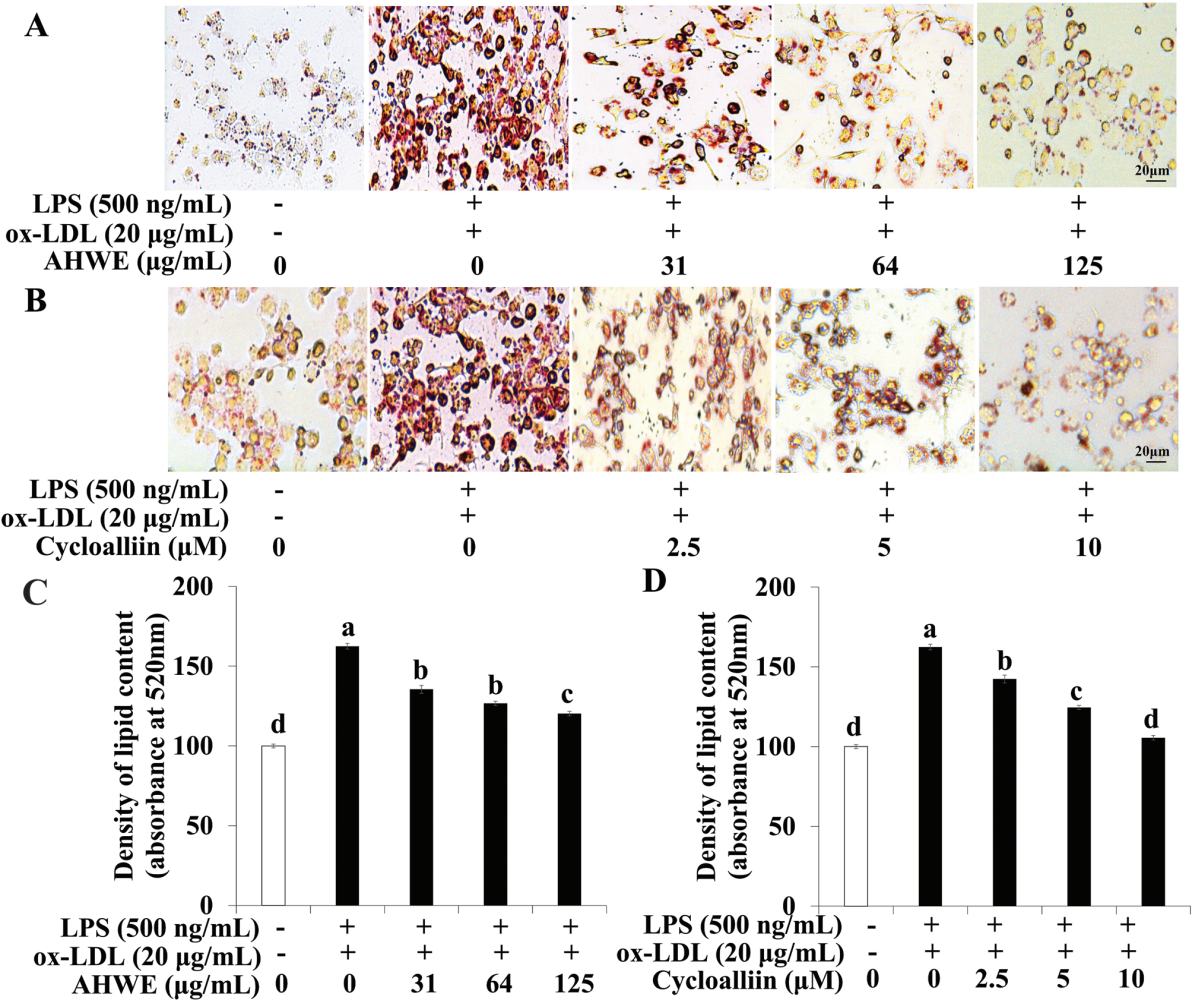
Downregulation of lipid accumulation by AHWE and cycloalliin treatment in THP-1 foam cells. THP-1 differentiated macrophages were cultured in the absence or presence of AHWE (0–125 μg/mL) and cycloalliin (0–10 μM) prior to 24 h. Subsequently, THP-1 cells were cultured in LPS (500 ng/mL) containing ox-LDL (20 μg/mL) for 24 h. (A–B) Cells were stained with Oil Red O; microphotographs were captured using an optical microscope at 400× magnification. (C–D) Stained cells were dissolved in an isopropanol solution, and the staining intensity was measured at 520 nm. LPS, lipopolysaccharides; ox-LDL, oxidized low-density lipoprotein; AHWE, *Allium hookeri* hot water extract; SD, Standard deviation.

The suppression of lipid accumulation and foam cell formation by AHWE and cycloalliin in THP-1 macrophages treated with ox-LDL and LPS co-treatment using Oil red O staining was evaluated. Figure 2A–D shows the strong red staining observed in macrophages co-treated with ox-LDL and LPS. Red staining indicates lipid accumulation degree. However, lipid accumulation significantly declined in macrophages exposed to AHWE (125 μg/mL) and cycloalliin (10 μM) (*P* < 0.05). These results showed that AHWE and cycloalliin inhibited the extent of lipoprotein accumulation and foam cell formation in macrophages co-treated with ox-LDL and LPS.

### AHWE and cycloalliin effects on lipid receptor expression in foam cells

**Fig. 3:**
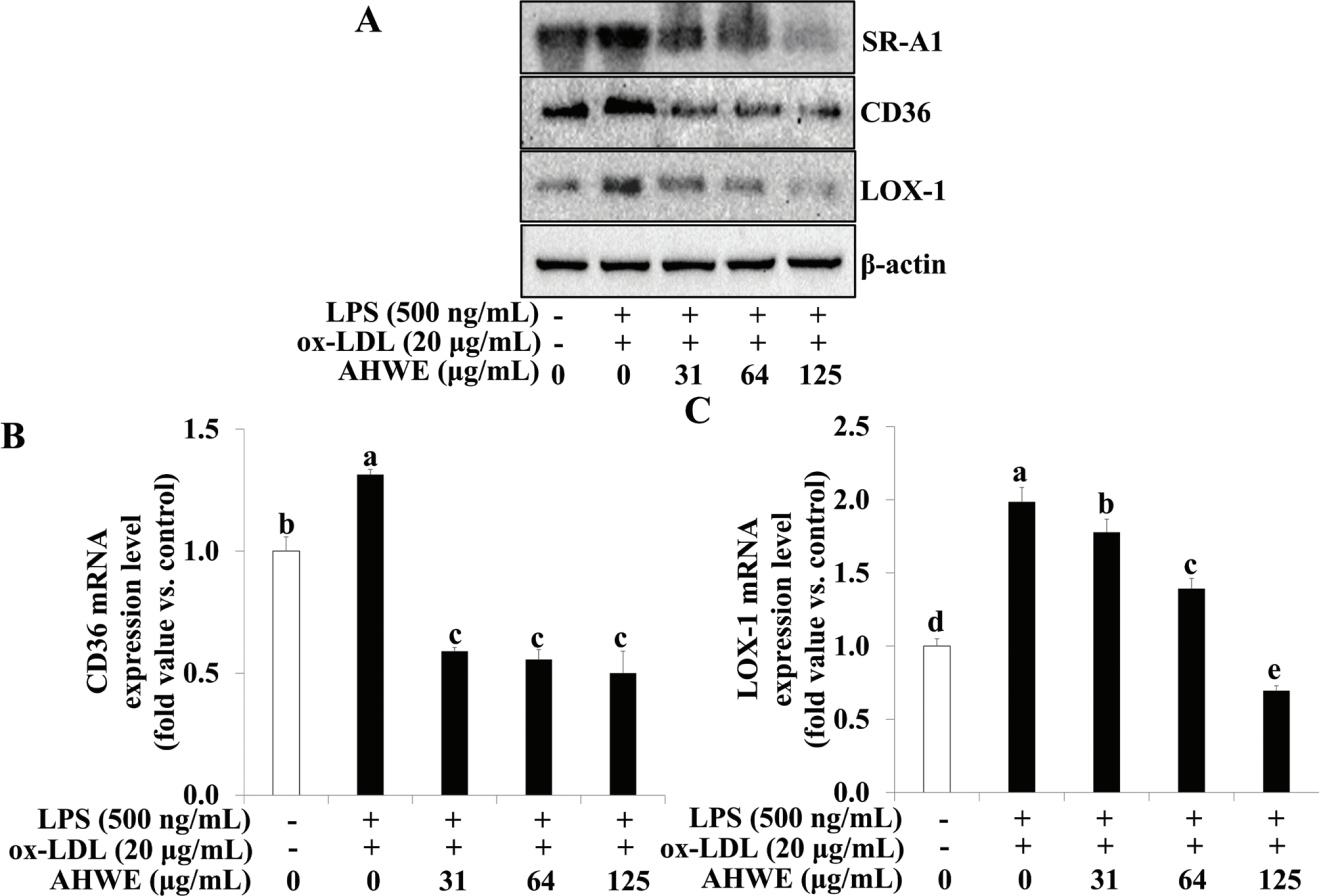
Inhibition of SR-A1, CD36, and LOX-1 expression by AHWE treatment in THP-1 foam cells. (A) The protein expression levels of SR-A1, CD36, and LOX-1 were assessed using immunoblotting. (B–C) Relative mRNA expression levels are shown after normalization against β-actin mRNA expression. (B) CD36 and (C) LOX-1 levels. The data are presented relative to the mRNA levels found in untreated cells, which were arbitrarily defined as 1. Experiments were performed in triplicate, and the results are presented as mean ± SD. Data analysis was performed using the 2^-∆∆CT^ method. Significant differences (*P* < 0.05) were identified using Duncan’s multiple range test, with distinct letters indicating significance. LPS, lipopolysaccharides; ox-LDL, oxidized low-density lipoprotein; AHWE, *Allium hookeri* hot water extract; SR-A1, scavenger receptor class A1; CD36, cluster of differentiation 36; LOX-1, lectin-like oxidized low-density lipoprotein receptor-1; SD, Standard deviation.

**Fig. 4:**
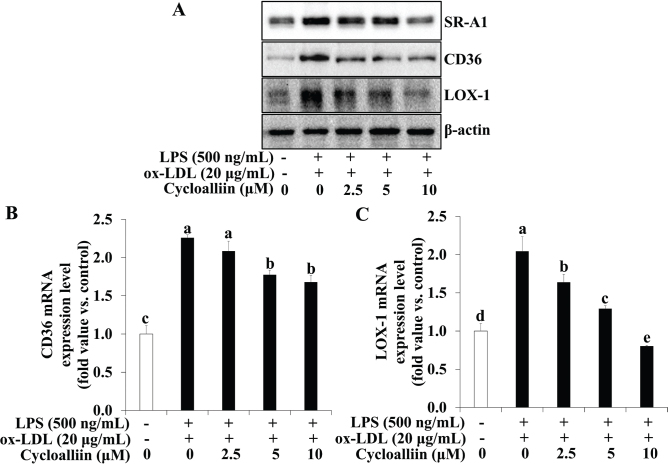
Inhibition of SR-A1, CD36, and LOX-1 expression via cycloalliin treatment in THP-1 foam cells. (A) Protein levels of SR-A1, CD36, and LOX-1 were assessed via immunoblotting. (B–C) The relative mRNA expression levels are shown after normalization against β-actin mRNA expression. (B) CD36 and (C) LOX-1 levels. The results are presented relative to the mRNA levels found in untreated cells, which was arbitrarily defined as 1. Experiments were performed in triplicate, and data are presented as mean ± SD. Analysis was performed using the 2^-∆∆CT^ method. Significance (*P* < 0.05) was determined through Duncan’s multiple range test, with different letters indicating significant differences. LPS, lipopolysaccharides; ox-LDL, oxidized low-density lipoprotein; SR-A1, scavenger receptor class A1; CD36, cluster of differentiation 36; LOX-1, lectin-like oxidized low-density lipoprotein receptor-1; SD, Standard deviation.

To explore the influence of AHWE and cycloalliin on reduced lipid accumulation in macrophages subjected to co-treatment with ox-LDL and LPS, the expression levels of CD36, SR-A1, and LOX-1 using immunoblotting and qPCR were determined. The expression of CD36, SR-A1*,* and LOX-1 increased significantly in macrophages co-treated with ox-LDL and LPS compared to those in untreated cells. However, pretreatment with AHWE and cycloalliin in ox-LDL and LPS co-treated THP-1 macrophages resulted in significantly reduced expression of CD36, SR-A1, and LOX-1 (*P* < 0.05) (Figs. 3A and 4A). Furthermore, the mRNA levels of *CD36* and *LOX-1* were increased in cells exposed to ox-LDL and LPS co-treatment compared to untreated cells, whereas treatment with AHWE and cycloalliin significantly decreased these levels (*P* < 0.05) (Figs. 3B–C and 4B–C). These results showed that AHWE and cycloalliin mitigate foam cell formation by suppressing lipid accumulation via the CD36/LOX-1/SR-A1 pathway.

### Effect of AHWE and cycloalliin on cholesterol efflux in foam cells

**Fig. 5:**
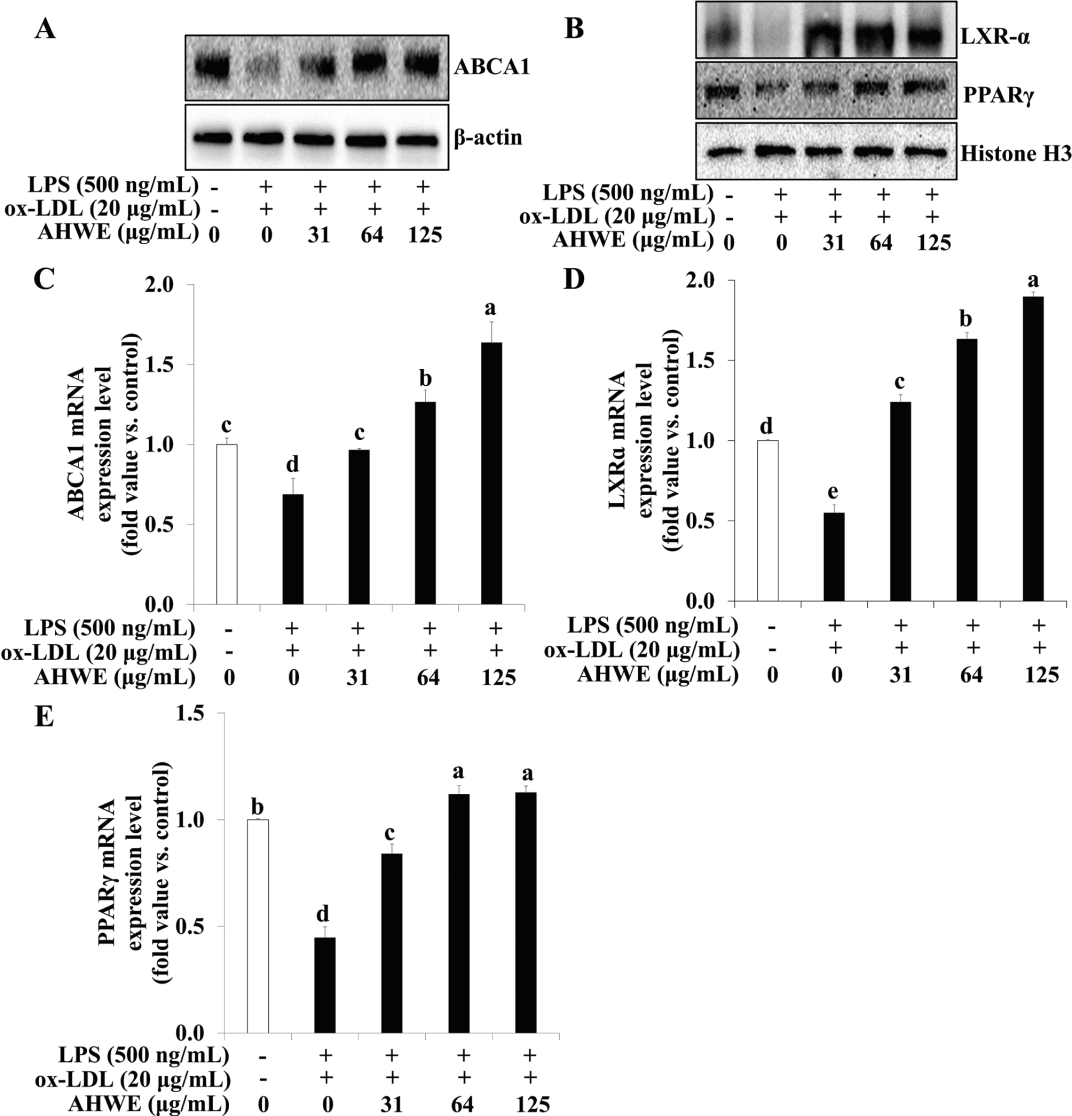
Upregulation of ABCA1, PPARγ, and LXR-α expression via AHWE treatment in THP-1 foam cells. The protein expression levels of ABCA1, LXRɑ, and PPARɣ were assessed using (A–B) immunoblotting. For mRNA expression, cells were harvested from ox-LDL and LPS-induced foam cells, and the levels of (C–E) ABCA1, LXRɑ, and PPARɣ mRNA were evaluated. (C) ABCA1, (D) LXRɑ, and (E) PPARɣ levels were quantified. Data are presented as the means ± SD. Analysis was performed using the 2^-∆∆CT^ method. Significant differences (*P* < 0.05) were determined through Duncan’s multiple range test, with different letters indicating significant differences. LPS, lipopolysaccharides; ox-LDL, oxidized low-density lipoprotein; AHWE, *Allium hookeri* hot water extract; ABCA1, ATP binding cassette transporter A1; LXRα, liver X receptor α; PPARɣ, Peroxisome proliferator-activated receptor γ; SD, Standard deviation.

**Fig. 6:**
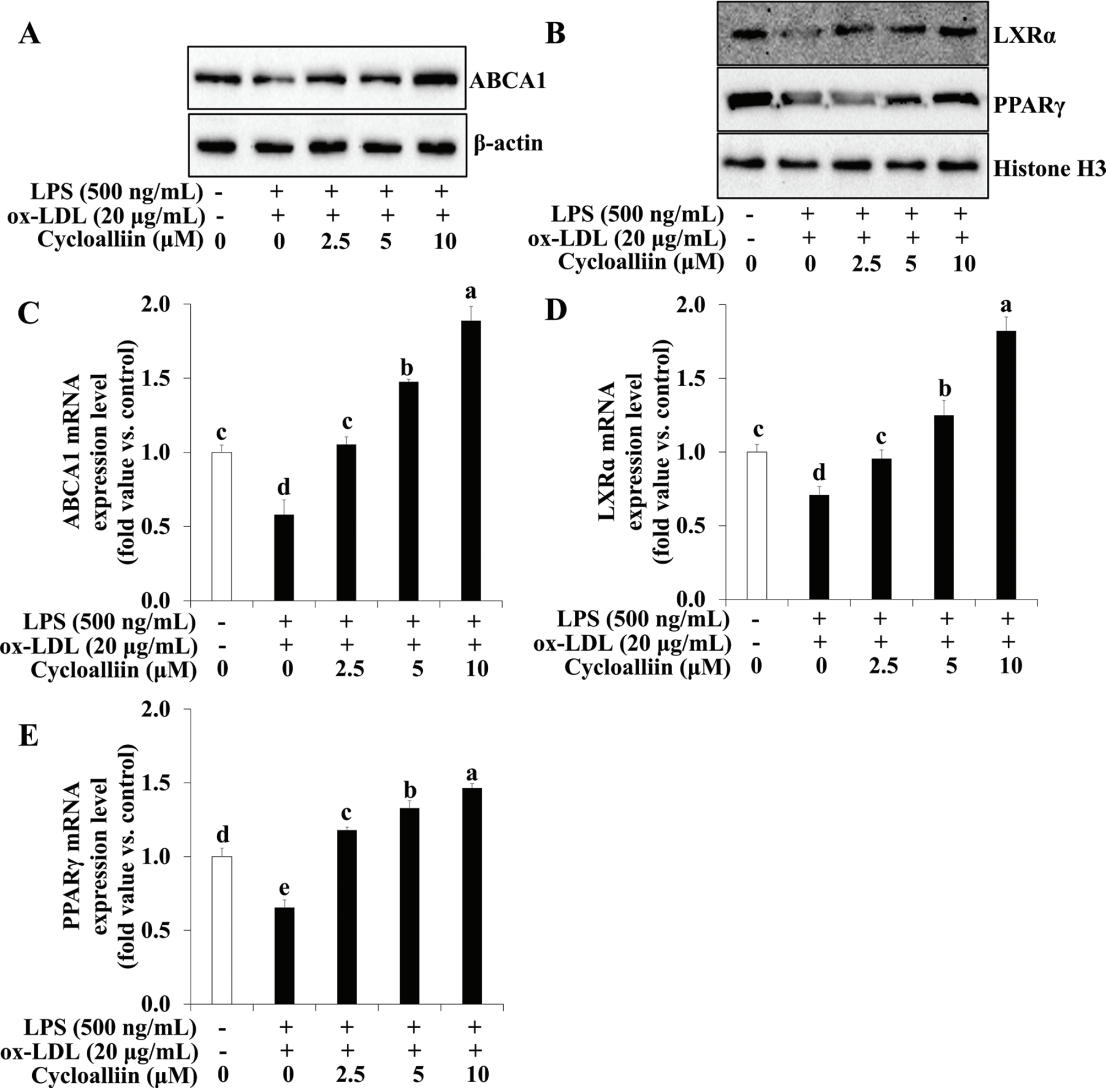
Upregulation of ABCA1, PPARγ, and LXR-α expression by cycloalliin treatment in THP-1 foam cells. (A–B) The protein expression levels of ABCA1, LXRɑ, and PPARɣ were assessed using immunoblotting. For mRNA expression analysis, cells from ox-LDL and LPS-induced foam cells were harvested, and the levels of (C–E) ABCA1, LXRɑ, and PPARɣ mRNA were evaluated. (C) ABCA1, (D) LXRɑ, and (E) PPARɣ levels. Data are presented as the means ± SD. Analysis was performed using the 2^-∆∆CT^ method. Significant differences (*P* < 0.05) were identified using Duncan’s multiple range test, with different letters indicating significant differences. LPS, lipopolysaccharides; ox-LDL, oxidized low-density lipoprotein; ABCA1, ATP binding cassette transporter A1; LXRα, liver X receptor α; PPARɣ, Peroxisome proliferator-activated receptor γ; SD, Standard deviation.

AHWE and cycloalliin effects on cellular cholesterol efflux in foam cells were investigated. Figures 5A–B and 6A–B show that co-treatment with ox-LDL and LPS decreased PPARγ, LXRα, and ABCA1 levels. However, treatment with AHWE and cycloalliin reversed this effect, resulting in increased levels of PPARγ, LXRα, and ABCA1. Additionally, the mRNA levels of PPARγ, LXRα, and ABCA1 were reduced in cells subjected to ox-LDL and LPS co-treatment compared to those in untreated cells; however, they significantly increased upon treatment with AHWE and cycloalliin (*P* < 0.05) (Figs. 5C–E and 6C–E). These findings suggest that AHWE and cycloalliin may play a pivotal role in enhancing cholesterol efflux, thereby preventing foam cell formation induced by ox-LDL and LPS.

### AHWE and cycloalliin effects on pro-inflammatory cytokine release and related gene expression via NF-κB pathway in foam cells

**Fig. 7:**
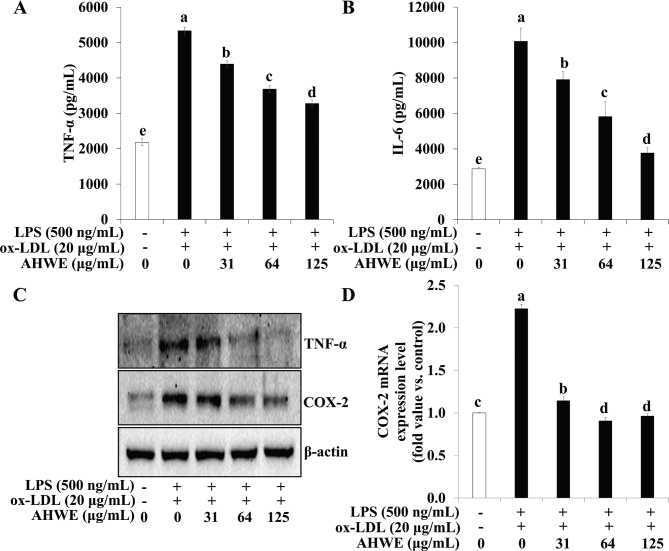
Inhibition of inflammatory cytokine secretion via AHWE treatment in co-treatment with ox-LDL and LPS-induced foam cell. THP-1 foam cells were pretreated with various concentrations of AHWE (0–125 μM) for 48 h. (A–B) The secretion of IL-6 and TNF-α was quantified using an ELISA kit. (C) Immunoblotting was employed to measure the expression levels of TNF-ɑ and COX-2. For mRNA expression analysis, cells from ox-LDL and LPS-induced foam cells were harvested, and the expression of (D) COX-2 mRNA was evaluated. Data are presented as the means ± SD. Statistical significance (*P* < 0.05) was determined using Duncan’s multiple range test, with different letters indicating significant differences. LPS, lipopolysaccharides; ox-LDL, oxidized low-density lipoprotein; AHWE, *Allium hookeri* hot water extract; TNF-α, tumor necrosis factor-α; IL-6, Interleukin 6; COX-2, Cyclooxygenase-2; ELISA, enzyme-linked immunosorbent assay; SD, Standard deviation.

**Fig. 8:**
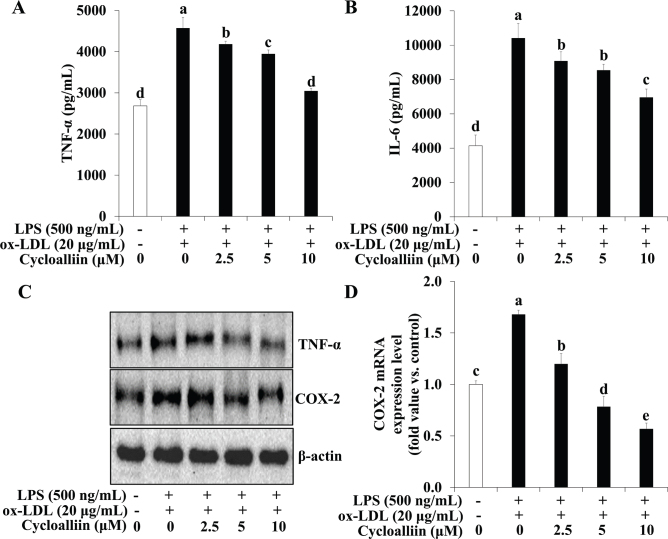
Inhibition of inflammatory cytokine secretion through cycloalliin treatment in co-treatment with ox-LDL and LPS-induced foam cell. THP-1 foam cells were pretreated with various concentrations of cycloalliin (0–10 μM) for 48 h. (A–B) The secretion of IL-6 and TNF-α was quantified using an ELISA kit. (C) Immunoblotting was used to measure the expression levels of TNF-ɑ and COX-2. For mRNA expression analysis, cells from ox-LDL and LPS-induced foam cells were harvested, and the expression of the (D) COX-2 mRNA was evaluated. Data are presented as the means ± SD. Statistical significance (*P* < 0.05) was determined using Duncan’s multiple range test, with different letters indicating significant differences. LPS, lipopolysaccharides; ox-LDL, oxidized low-density lipoprotein; TNF-α, tumor necrosis factor-α; IL-6, Interleukin 6; COX-2, Cyclooxygenase-2; ELISA, enzyme-linked immunosorbent assay; SD, Standard deviation.

**Fig. 9:**
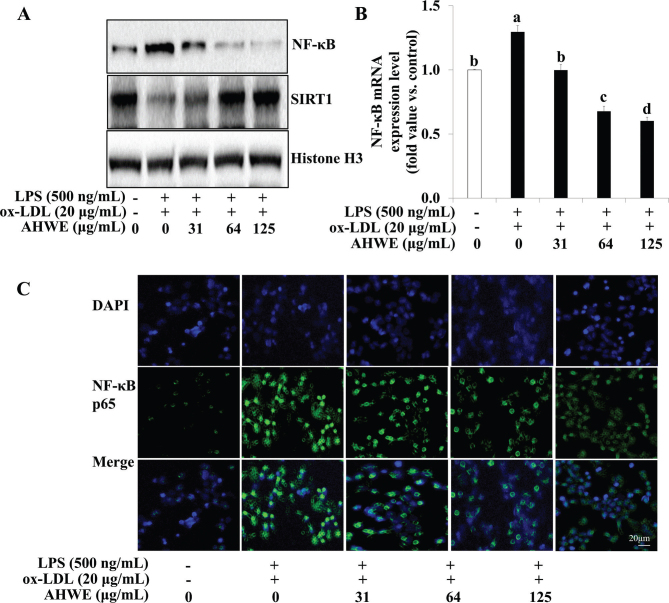
Inhibition of NF-κB p65 activation via AHWE treatment in co-treatment with ox-LDL and LPS-induced foam cell. (A) The levels of the NF-κB and SIRT1 proteins were assessed using immunoblotting. For mRNA expression analysis, cells from ox-LDL and LPS-induced foam cells were harvested, and the expression of the (B) NF-κB mRNA was evaluated. Data are presented as the means ± SD. Statistical significance (*P* < 0.05) was determined using Duncan’s multiple range test, with different letters indicating significant differences. (C) THP-1 foam cells were treated with AHWE (0–125 μg/mL) and then fixed with 4% PFA. After blocking with an appropriate buffer, cells were incubated with antibodies. Subsequently, DAPI staining was performed to confirm the nuclei in the cells. The signals were quantified using fluorescence microscopy at 400× magnification. LPS, lipopolysaccharides; ox-LDL, oxidized low-density lipoprotein; AHWE, *Allium hookeri* hot water extract; NF-κB, nuclear factor-κB; SIRT1, sirtuin 1; DAPI, 4’,6-diamidino-2-phenylindole; SD, Standard deviation.

**Fig. 10:**
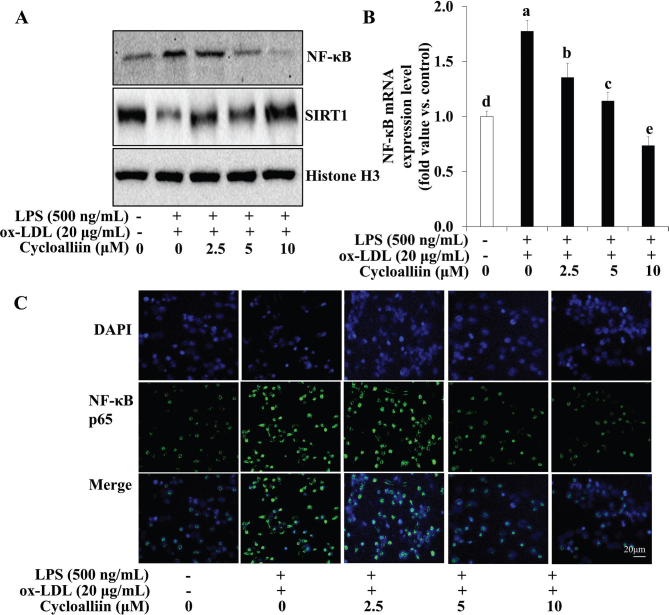
Inhibition of NF-κB p65 activation by cycloalliin treatment in co-treatment with ox-LDL and LPS-induced foam cell. (A) The protein levels of NF-κB and SIRT1 were assessed using immunoblotting. For mRNA expression analysis, cells from ox-LDL and LPS-induced foam cells were harvested, and the expression of the (B) NF-κB mRNA was evaluated. Data are presented as the means ± SD. Statistical significance (*P* < 0.05) was determined using Duncan’s multiple range test, with different letters indicating significant differences. (C) THP-1 foam cells were treated with cycloalliin (0–10 μM) and then fixed with 4% PFA. After blocking with an appropriate buffer, cells were incubated with antibodies. Subsequently, DAPI staining was performed to confirm the nuclei in the cells. The signals were quantified using fluorescence microscopy at 400× magnification. LPS, lipopolysaccharides; ox-LDL, oxidized low-density lipoprotein; NF-κB, nuclear factor-κB; SIRT1, sirtuin 1; DAPI, 4’,6-diamidino-2-phenylindole; SD, Standard deviation.

Proinflammatory cytokines secretion and NF-κB expression were examined following AHWE and cycloalliin treatment in combined ox-LDL and LPS-induced foam cell formation. Figures 7A–B and 8A–B show that ELISA assays revealed a significant increase in the secretion of the inflammatory cytokines IL-6 and TNF-α during foam cell formation, with AHWE and cycloalliin effectively suppressing this overproduction of cytokines (*P* < 0.05). Conversely, an increase was observed in the expression of proteins COX-2 and TNF-α in foam cell formation induced by combined ox-LDL and LPS (Figs. 7C–D and 8C–D). However, AHWE and cycloalliin downregulated the expression of COX-2 and TNF-α. NF-κB activation influences multiple stages of atherosclerosis, from plaque formation to vascular rupture (44). Particularly in the early stages, NF-κB serves as a mediator for the expression of inflammatory genes and facilitates the transformation of macrophages into foam cells (45). Figures 9A and 10A show that AHWE and cycloalliin treatment significantly lowered NF-κB expression (*P* < 0.05). Additionally, AHWE and cycloalliin significantly decreased the mRNA expression level of the NF-κB gene in foam cells compared to those in the ox-LDL and LPS co-treatment control (*P* < 0.05) (Figs. 9B and 10B). Immunofluorescence analysis further demonstrated the inhibitory effect of 125 μg/mL AHWE and 10 μM cycloalliin on ox-LDL and LPS-induced nuclear translocation of p65 (Figs. 9C and 10C). These findings suggest that AHWE and cycloalliin may serve as potential inhibitors of inflammation by reducing inflammation in foam cells during the early stages of atherosclerosis.

## Discussion

Cardiovascular diseases stand as a leading cause of death and disability globally (46). The World Health Organization reported that cardiovascular disease accounted for 32% of global mortality in 2019 (47). According to data from the 2019 Korea National Health and Nutrition Examination Survey, the prevalence of cardiovascular diseases in South Korea was 21.8% (48). This prevalence seems to rise with age, starting from the age of 50 years (49). Atherosclerosis, a crucial cause of cardiovascular disease, is characterized by foam cell formation after macrophages phagocytose ox-LDL in the early stage (8). Accumulation of lipid-containing foam cells accelerates plaque formation owing to abnormal cholesterol metabolism and increased inflammation (50). Therefore, regulating the balance of cholesterol inflow and outflow to prevent lipid accumulation within macrophages and inhibit their transformation into foam cells becomes a significant factor in preventing and treating atherogenesis (7). Research into natural dietary agents, including quercetin, berberine, and curcumin, is focused on inhibiting foam cell formation and promoting cholesterol efflux (51). *A. hookeri,* a plant from the Alliaceae family, contains sulfur and phenolic compounds, flavonoids, and allicin (32). It is primarily used not only for traditional medicinal purposes, including promoting digestive health and combating inflammation but also as a culinary component in Asian cuisine (32, 33). A recent analysis of the marker component cycloalliin in a freeze-dried sample of *A. hookeri* root water extract revealed that cycloalliin was present at 5.44%. Cycloalliin, a sulfur-containing cyclic compound, has physiological activities such as antioxidant, antithrombotic, and antiviral effects (41).

Currently, atherosclerosis is a common disease characterized by the accumulation of fatty deposits known as atheromatous plaques within the inner layers of arteries (7). In the early stages of atherosclerosis, LDL levels serve as an indicator of atherosclerosis development, with elevated serum LDL concentrations widely recognized as a primary risk factor for coronary atherosclerosis. Fuenzalida identified the main mechanisms driving macrophage formation as the uptake of oxidized LDL and impaired lipid efflux (52). Macrophages, pivotal players in atherosclerosis development, become activated subendothelially within atherogenic lesions after ingesting LDL (7). Monocytes recruited to vascular plaques undergo differentiation into macrophages upon infiltration, subsequently transforming into foam cells when overloaded with ox-LDL, marking a critical step in atherosclerosis progression (3, 4). Foam cells, integral components of atherosclerotic plaques, significantly contribute to the onset of atherosclerosis, eventually accelerating the core formation of fibrous atheromas (12, 13). The transformation of macrophages into foam cells triggers an excessive inflammatory response in atherosclerosis pathogenesis (22). Clinical studies have observed heightened levels of inflammatory cytokines in the serum of patients with atherosclerosis (53). Furthermore, ox-LDL, with its cytotoxic effects, induces the expression of inflammatory genes, thereby promoting foam cell formation. Pro-inflammatory cytokines, including TNF-α, COX-2, and IL-6, recruit monocytes to the vessel wall, augment ox-LDL uptake, and enhance SR expression, thereby accelerating foam cell formation and atherosclerosis (26–28). Therefore, the mechanism of suppressing foam cell formation in the early stages of arteriosclerosis emerges as a crucial event not only in preventing arteriosclerosis but also cardiovascular disease. These findings revealed that the combined treatment of ox-LDL and LPS led to an increase in intracellular lipid accumulation, evidenced by the heightened presence of Oil-red O-stained lipid particles within cells. This data indicates that concentrations of AHWE at 125 μg/mL and cycloalliin at 10 μM significantly mitigated lipid droplet formation compared to those in untreated cells. Consistent with this finding, Im et al. reported that PEITC inhibited the formation of foam cells by LPS and ox-LDL in THP-1 foam cells, as observed through Oil red O staining (54). Fu et al. reported that sodium paeonolsilate reduced lipid accumulation in ox-LDL-induced THP-1 foam cells, also assessed by Oil red O staining (55).

Numerous studies have highlighted the pivotal role of various cytokines in driving the progression of atherosclerosis and plaque instability (22). IL-6, produced by leukocytes and other cells, serves as a key regulator of inflammatory responses and is recognized as a biomarker of inflammation (56). TNF-α, known for its potent pro-inflammatory effects, not only induces the expression of other inflammatory cytokines and adhesion molecules but also triggers apoptosis of vascular smooth muscle cells, thereby promoting atherosclerosis and plaque instability (57). NF-κB is the main signaling pathway that influences foam cell aggregation and inflammatory response (45). Research by Tong et al. demonstrated that resveratrol suppressed inflammation through the MAPK/NF-κB signaling pathway in LPS-induced foam cells (58). Nguyen et al. found that *Lasia spinosa* Leaf Extract inhibited TNF-α, PGE2, COX-2, and NO production through the MAPK and NF-κB pathway in LPS-stimulated foam cells (59). In this study, it was observed that an elevation in the protein expression of NF-κB, TNF-α, and COX-2, along with a decrease in SIRT1 expression, following co-treatment with LPS and ox-LDL compared to those in untreated cells. In contrast, treatment with AHWE and cycloalliin resulted in the downregulation of COX-2, TNF-α, and NF-κB expression, coupled with an upregulation of SIRT1. These findings suggest that AHWE and cycloalliin jointly inhibit proinflammatory events by modulating NF-κB expression and inhibiting cytokine release in co-treated ox-LDL and LPS-induced foam cells.

An imbalance between cholesterol efflux and uptake leads to the formation of macrophage-derived foam cells (60), a critical event in the early stages of atherosclerotic lesion development. Therefore, regulating the cholesterol mechanism is essential for preventing foam cell formation. Cholesterol clearance from macrophages is crucial for preventing the formation of foam cells, which are a characteristic feature of the initial phases of atherosclerotic lesion development. ABCA1 plays a major role in regulating reverse cholesterol transport (19). It facilitates cholesterol efflux, converting cholesterol and phospholipids into HDL lipoproteins, specifically apoA-1. This process inhibits the formation of foam cells derived from macrophages, thereby preventing atherosclerosis (61). Cell studies and experiments with ABCA1 transgenic mice have demonstrated that reducing ABCA1 expression leads to decreased cholesterol efflux and increased lipid accumulation. The nuclear receptor LXR serves as a cholesterol sensor and activates proteins involved in reverse cholesterol transport, such as those responsible for cholesterol transport and uptake (62). LXR activation increases HDL formation via ABCA1 and promotes cholesterol efflux and intestinal cholesterol excretion (63). LXR-deficient mice demonstrate accelerated atherosclerosis owing to accumulating cholesterol, whereas curcumin-fed mice, which activate LXRα, show inhibition of atherosclerosis development (62). LXRα induces ABCA1 expression and facilitates cholesterol clearance from macrophages, mediated by PPARγ. The transcription factor PPARγ, involved in adipogenesis and lipid metabolism, has been implicated in metabolic diseases such as cardiovascular and chronic kidney diseases (20). During foam cell formation, cholesterol intake and reserve cholesterol transport play crucial roles. Cholesterol intake is mediated by specific proteins, including PPARɣ and SIRT1. SIRT1, an upstream regulator of PPARɣ, modulates the cholesterol efflux pathway. Furthermore, PPARγ activation is suggested to promote adipocyte differentiation while simultaneously suppressing the expression of inflammatory genes, including TNF-α and IL-1β, in macrophages. It suggests that the nuclear receptor PPARγ and LXRα may cooperate in regulating ABCA1 expression to promote cholesterol clearance in macrophages. Li et al. reported that quercetin regulates LXRα and ABCA1 expression in ox-LDL-treated RAW 264.7 cells (64), while Nyandwi et al. demonstrated that rosmarinic acid increases cholesterol efflux via ABCA1 in ox-LDL-treated THP-1 cells (65). Additionally, Li et al. found that baicalei enhances cholesterol efflux through the CD36/JNK/ABCA1 signaling pathway (66).

In atherosclerotic plaques, macrophages mediate the uptake of ox-LDL through cell surface receptors SR-A1, LOX-1, and CD36 (12, 13). Previous studies have associated lipid uptake with SRs. Excessive activation of LOX-1, primarily expressed in vascular endothelial cells, is associated with vascular disease development, potentially compromising endothelial cell survival and function (67). LOX-1 levels rise under conditions of oxidative stress, contributing to atherosclerosis by capturing oxidized LDL and triggering an inflammatory response in vascular endothelial cells (68). Various pro-atherogenic cytokines, including TNF-α, IL-1β, and IFNγ, stimulate the expression of cellular LOX-1 (69). A recent study demonstrated that reducing LOX-1 expression inhibits ox-LDL-induced foam cell formation and atherosclerosis (70). CD36, a protein found on the surface of macrophages, adipocytes, and liver cells, plays a crucial role in the cellular uptake of fatty acids. Macrophages utilize CD36 to uptake ox-LDL (71). The interaction between CD36 and ox-LDL triggers cytokine secretion (72). According to Kuchibhotla et al., macrophages in CD36-null mice demonstrate impaired ox-LDL uptake, leading to reduced atherosclerotic lesion formation (73). Upregulation of SR-A and CD36 expression promotes ox-LDL uptake, leading to arterial inflammation characterized by cytokine secretion and foam cell formation in the artery intima (74). Duan et al. reported that tetramethylpyrazine inhibits lipid accumulation in macrophages by regulating the SR-A/CD36 signaling pathway (75). Lin et al. found that andrographolide extracted from *Andrographis paniculata* reduces CD36 levels in J774A.1 cells, thereby attenuating ox-LDL-mediated foam cell formation (76). Chen et al. reported that polyphenolics from *Syzygium brachythyrsum* suppress foam cell formation by decreasing CD36 and SR-A1 levels in ox-LDL-mediated murine macrophages (77).

Therefore, the ABCA1/LXR/PPARɣ mechanism, which facilitates cholesterol efflux, and the CD36/LOX-1/SR-A1 mechanism, which contributes to lipid accumulation, as pathways that inhibit foam cell formation were focused on. Consistent with findings from previous studies, AHWE and cycloalliin were found to improve cholesterol efflux in ox-LDL-loaded macrophages by upregulating ABCA1/LXRα/PPARɣ expression and simultaneously suppressing lipid uptake by increasing levels of SR-A1, CD36, and LOX-1 in THP-1 macrophages.

## Conclusions

Generally, AHWE and cycloalliin effectively inhibited foam cell formation by regulating lipid accumulation and promoting cholesterol efflux in foam cells induced by a combined treatment of ox-LDL and LPS. Additionally, AHWE and cycloalliin suppressed the production of inflammatory cytokines and the expression of NF-κB and its target genes in foam cells. Collectively, these findings underscore the effectiveness of AHWE and cycloalliin as natural substances for preventing and treating atherosclerosis.

## References

[1] Kim TH, Son YK, Hwang KH, Kim MH. Effects of Angelica keiskei Koidzumi and turmeric extract supplementation on serum lipid parameters in hypercholesterolemic diet or P-407-induced hyperlipidemic rats. J Korean Sco Food Sci Nutr 2008; 37(6): 708–13. doi: 10.3746/jkfn.2008.37.6.708

[2] Chi L, Peng L, Pan N, Hu X, Zhang Y. The anti-atherogenic effects of berberine on foam cell formation are mediated through the upregulation of sirtuin 1. Int J Mol Med 2014; 34(4): 1087–93. doi: 10.3892/ijmm.2014.1868 25069720

[3] Hansson GK. Inflammation, atherosclerosis, and coronary artery disease. N Engl J Med 2005; 352(16): 1685–95. doi: 10.1056/NEJMra043430 15843671

[4] Weber C, Zernecke A, Libby P. The multifaceted contributions of leukocyte subsets to atherosclerosis: lessons from mouse models. Nat Rev Immunol 2008; 8(10): 802–15. doi: 10.1038/nri2415 18825131

[5] Cybulsky MI, Gimbrone Jr MA. Endothelial expression of a mononuclear leukocyte adhesion molecule during atherogenesis. Science 1991; 251(4995): 788–91. doi: 10.1126/science.1990440 1990440

[6] Yoshida H, Kisugi R. Mechanisms of LDL oxidation. Clin Chim Acta 2010; 411(23–24): 1875–82. doi: 10.1016/j.cca.2010.08.038 20816951

[7] Moore KJ, Sheedy FJ, Fisher EA. Macrophages in atherosclerosis: a dynamic balance. Nat Rev Immunol 2013; 13(10): 709–21. doi: 10.1038/nri3520 23995626 PMC4357520

[8] Yu XH, Fu YC, Zhang DW, Yin K, Tang CK. Foam cells in atherosclerosis. Clin Chim Acta 2013; 424: 245–52. doi: 10.1016/j.cca.2013.06.006 23782937

[9] Voloshyna I, Hai O, Littlefield MJ, Carsons S, Reiss AB. Resveratrol mediates anti-atherogenic effects on cholesterol flux in human macrophages and endothelium via PPARγ and adenosine. Eur J Pharmacol 2013; 698(1–3): 299–309. doi: 10.1016/j.ejphar.2012.08.024 23041272

[10] Tabas I, García-Cardeña G, Owens GK. Recent insights into the cellular biology of atherosclerosis. J Cell Biol 2015; 209(1): 13–22. doi: 10.1083/jcb.201412052 25869663 PMC4395483

[11] Cai Y, Li JD, Yan C. Vinpocetine attenuates lipid accumulation and atherosclerosis formation. Biochem Biophys Res Commun 2013; 434(3): 439–43. doi: 10.1016/j.bbrc.2013.03.092 23583194 PMC3682500

[12] Park YM, Febbraio M, Silverstein RL. CD36 modulates migration of mouse and human macrophages in response to oxidized LDL and may contribute to macrophage trapping in the arterial intima. J Clin Invest 2009; 119(1): 136–45. doi: 10.1172/JCI35535 19065049 PMC2613464

[13] Sukhorukov VN, Khotina VA, Chegodaev YS, Ivanova E, Sobenin IA, Orekhov AN. Lipid metabolism in macrophages: focus on atherosclerosis. Biomedicines 2020; 8(8): 262. doi: 10.3390/biomedicines8080262 32752275 PMC7459513

[14] Leifer CA, Gruber E, Stelzer S, Erlich E, Sinha S. Macrophage lipid accumulation is regulated by substrate stiffness. J Immunol 2016; 196(1_Suppl): 57.5. doi: 10.4049/jimmunol.196.Supp.57.5

[15] Ouimet M, Barrett TJ, Fisher EA. HDL and reverse cholesterol transport: basic mechanisms and their roles in vascular health and disease. Circ Res 2019; 124(10): 1505–18. doi: 10.1161/CIRCRESAHA.119.312617 31071007 PMC6813799

[16] Westerterp M, Murphy AJ, Wang M, Pagler TA, Vengrenyuk Y, Kappus MS, et al. Deficiency of ATP-binding cassette transporters A1 and G1 in macrophages increases inflammation and accelerates atherosclerosis in mice. Circ Res 2013; 112(11): 1456–65. doi: 10.1161/CIRCRESAHA.113.301086 23572498 PMC3839866

[17] Westerterp M, Fotakis P, Ouimet M, Bochem AE, Zhang H, Molusky MM, et al. Cholesterol efflux pathways suppress inflammasome activation, NETosis, and atherogenesis. Circulation 2018; 138(9): 898–912. doi: 10.1161/CIRCULATIONAHA.117.032636 29588315 PMC6160368

[18] Shea S, Stein JH, Jorgensen NW, McClelland RL, Tascau L, Shrager S, et al. Cholesterol mass efflux capacity, incident cardiovascular disease, and progression of carotid plaque: the multi-ethnic study of atherosclerosis. Arterioscler Thromb Vasc Biol 2019; 39(1): 89–96. doi: 10.1161/ATVBAHA.118.311366 30580560 PMC6310062

[19] Zhang Y, Igwe OJ. Exogenous oxidants activate nuclear factor kappa B through Toll-like receptor 4 stimulation to maintain inflammatory phenotype in macrophage. Biochem Pharmacol 2018; 147: 104–18. doi: 10.1016/j.bcp.2017.11.012 29175419 PMC5733690

[20] Venkateswaran A, Laffitte BA, Joseph SB, Mak PA, Wilpitz DC, Edwards PA, et al. Control of cellular cholesterol efflux by the nuclear oxysterol receptor LXRα. Proc Natl Acad Sci U S A 2000; 97(22): 12097–102. doi: 10.1073/pnas.200367697 11035776 PMC17300

[21] Chawla A, Boisvert WA, Lee C-H, Laffitte BA, Barak Y, Joseph SB, et al. A PPARγ-LXR-ABCA1 pathway in macrophages is involved in cholesterol efflux and atherogenesis. Mol Cell 2001; 7(1): 161–71. doi: 10.1016/s1097-2765(01)00164-2 11172721

[22] Malekmohammad K, Sewell RD, Rafieian-Kopaei M. Antioxidants and atherosclerosis: mechanistic aspects. Biomolecules 2019; 9(8): 301. doi: 10.3390/biom9080301 31349600 PMC6722928

[23] Sprague AH, Khalil RA. Inflammatory cytokines in vascular dysfunction and vascular disease. Biochem Pharmacol 2009; 78(6): 539–52. doi: 10.1016/j.bcp.2009.04.029 19413999 PMC2730638

[24] Kim KW, Ivanov S, Williams JW. Monocyte recruitment, specification, and function in atherosclerosis. Cells 2020; 10(1): 15. doi: 10.3390/cells10010015 33374145 PMC7823291

[25] Kong P, Cui ZY, Huang XF, Zhang DD, Guo RJ, Han M. Inflammation and atherosclerosis: signaling pathways and therapeutic intervention. Signal Transduct Target Ther 2022; 7(1): 131. doi: 10.1038/s41392-022-00955-7 35459215 PMC9033871

[26] Makarov SS. NF-κB in rheumatoid arthritis: a pivotal regulator of inflammation, hyperplasia, and tissue destruction. Arthritis Res 2001; 3(4): 1–7. doi: 10.1186/ar300 11438035 PMC128895

[27] Pamukcu B, Lip GY, Shantsila E. The nuclear factor–kappa B pathway in atherosclerosis: a potential therapeutic target for atherothrombotic vascular disease. Thromb Res 2011;1 28(2): 117–23. doi: 10.1016/j.thromres.2011.03.025 21636112

[28] Zhang JM, An J. Cytokines, inflammation and pain. Int Anesthesiol Clin 2007; 45(2): 27. doi: 10.1097/AIA.0b013e318034194e 17426506 PMC2785020

[29] Pfluger PT, Herranz D, Velasco-Miguel S, Serrano M, Tschöp MH. Sirt1 protects against high-fat diet-induced metabolic damage. Proc Natl Acad Sci U S A 2008; 105(28): 9793–8. doi: 10.1073/pnas.0802917105 18599449 PMC2474520

[30] Guyton JR, Bays HE, Grundy SM, Jacobson TA. An assessment by the Statin Intolerance Panel: 2014 update. J Clin Lipidol 2014; 8(3): S72–81. doi: 10.1016/j.jacl.2014.03.002 24793444

[31] Sung J, Kim SH, Song HR, Chi MH, Park JE. Lipid-lowering treatment practice patterns in Korea: comparison with the data obtained from the CEPHEUS Pan-Asian study. J Atheroscler Thromb 2014; 21(11): 1219–27. doi: 10.5551/jat.23242 25069813

[32] Ayam VS. *Allium hookeri*, Thw. Enum. A lesser known terrestrial perennial herb used as food and its ethnobotanical relevance in Manipur. AJFAND 2011; 11(6): 5389–12. doi: 10.18697/ajfand.47.9330

[33] Sharma G, Gohil R, Kaul V. Cytological status of Allium hookeri Thwaites (2n= 22). Genet Resour Crop Evol 2011; 58: 1041–50. doi: 10.1007/s10722-010-9641-x

[34] Lee KW, Kim YS, Park PJ, Jeong JH. Comparison of effect of water and ethanolic extract from roots and leaves of *Allium hookeri*. J Korean Soc Food Sci Nutr 2014; 43(12): 1808–16. doi: 10.3746/jkfn.2014.43.12.1808

[35] Park JY, Yoon KY. Comparison of the nutrient composition and quality of the root of *Allium hookeri* grown in Korea and Myanmar. Korean J Food Sci Technol 2014; 46(5): 544–8. doi: 10.9721/KJFST.2014.46.5.544

[36] Myint AA, Aregay MG, Kang M, Kim BS, Lee YW, Kim J. Comprehensive study on the formation mechanism of highly bioactive compounds from *Allium hookeri* root using subcritical water and their antioxidant and anticancer effects. J Supercrit Fluids 2020; 157: 104709. doi: 10.1016/j.supflu.2019.104709

[37] Bae GC, Bae DY. The anti-inflammatory effects of ethanol extract of *Allium hookeri* cultivated in South Korea. Kor J Herbol 2012; 27(6): 55–61. doi: 10.6116/kjh.2012.27.6.55

[38] Bok SH, Seo JH, Bae CS, Kang B, Cho SS, Park DH. Allium hookeri root extract regulates asthmatic changes through immunological modulation of Th1/Th2‑related factors in an ovalbumin‑induced asthma mouse model. Mol Med Rep 2019; 20(4): 3215–23. doi: 10.3892/mmr.2019.10560 31432168 PMC6755185

[39] Li R, Wang YF, Sun Q, Hu HB. Chemical composition and antimicrobial activity of the essential oil from *Allium hookeri* consumed in Xishuangbanna, southwest China. Nat Prod Commun 2014; 9(6): 863–4. doi: 10.1177/1934578X1400900636 25115101

[40] Kim S, Lee S, Shin D, Yoo M. Change in organosulfur compounds in onion (Allium cepa L.) during heat treatment. Food Sci Biotechnol 2016; 25(1): 115–9. doi: 10.1007/s10068-016-0017-7 30263245 PMC6049342

[41] Sciences NIoA. Investigation of *Allium hookeri*’s functional activity and its use in developing functional foods. Jeonju: Rural Development Administration; 2016. Report No.: PJ010490.

[42] Yanagita T, Han S, Wang YM, Tsuruta Y, Anno T. Cycloalliin, a cyclic sulfur imino acid, reduces serum triacylglycerol in rats. Nutrition 2003; 19(2): 140–3. doi: 10.1016/s0899-9007(02)00857-2 12591546

[43] Yoshinari O, Shiojima Y, Igarashi K. Anti-obesity effects of onion extract in Zucker diabetic fatty rats. Nutrients 2012; 4(10): 1518–26. doi: 10.3390/nu4101518 23201769 PMC3497009

[44] Morgan MJ, Liu Zg. Crosstalk of reactive oxygen species and NF-κB signaling. Cell Res 2011; 21(1): 103–15. doi: 10.1038/cr.2010.178 21187859 PMC3193400

[45] Yu XH, Zheng XL, Tang CK. Nuclear factor-κB activation as a pathological mechanism of lipid metabolism and atherosclerosis. Adv Clin Chem 2015; 70: 1–30. doi: 10.1016/bs.acc.2015.03.004 26231484

[46] Ha KH, Kwon HS, Kim DJ. Epidemiologic characteristics of dyslipidemia in Korea. J Lipid Atheroscler 2015; 4(2): 93–9. doi: 10.12997/jla.2015.4.2.93

[47] Kaptoge S, Pennells L, De Bacquer D, Cooney MT, Kavousi M, Stevens G, et al. World Health Organization cardiovascular disease risk charts: revised models to estimate risk in 21 global regions. Lancet Glob Health 2019; 7(10): e1332–e45. doi: 10.1016/S2214-109X(19)30318-3 31488387 PMC7025029

[48] Oh K, Kim Y, Kweon S, Kim S, Yun S, Park S, et al. Korea national health and nutrition examination survey, 20th anniversary: accomplishments and future directions. Epidemiol Health 2021; 43: e2021025. doi: 10.4178/epih.e2021025 33872484 PMC8289475

[49] Lee YH, Kwak EM, Jo M. Factors affecting cardiovascular disease in Korea adults: focusing on smoking behavior including Urine cotinine and health behaviors. J Converg Cult Technol 2021; 7(3): 293–301.

[50] Maguire EM, Pearce SWA, Xiao Q. Foam cell formation: a new target for fighting atherosclerosis and cardiovascular disease. Vascul Pharmacol 2019; 112: 54–71. doi: 10.1016/j.vph.2018.08.002 30115528

[51] Wang D, Yang Y, Lei Y, Tzvetkov NT, Liu X, Yeung AWK, et al. Targeting foam cell formation in atherosclerosis: therapeutic potential of natural products. Pharmacol Rev 2019; 71(4): 596–670. doi: 10.1124/pr.118.017178 31554644

[52] Fuenzalida B, Cantin C, Kallol S, Carvajal L, Pastén V, Contreras-Duarte S, et al. Cholesterol uptake and efflux are impaired in human trophoblast cells from pregnancies with maternal supraphysiological hypercholesterolemia. Sci Rep 2020; 10(1): 5264. doi: 10.1038/s41598-020-61629-4 32210256 PMC7093446

[53] Tousoulis D, Economou EK, Oikonomou E, Papageorgiou N, Siasos G, Latsios G, et al. The role and predictive value of cytokines in atherosclerosis and coronary artery disease. Curr Med Chem 2015; 22(22): 2636–50. doi: 10.2174/0929867322666150415145814 25876746

[54] Im YS, Gwon MH, Yun JM. Protective effects of phenethyl isothiocyanate on foam cell formation by combined treatment of oxidized low-density lipoprotein and lipopolysaccharide in THP-1 macrophage. Food Sci Nutr 2021; 9(6): 3269–79. doi: 10.1002/fsn3.2293 34136191 PMC8194743

[55] Fu W, Fu W, Ding S, Chen Z, Jiang J, Gong Z, et al. Sodium paeonolsilate inhibits ox-LDL induced macrophage foam cell formation and inflammation in atherosclerosis. Int J Clin Exp Med 2016; 9(2): 1051–61.

[56] Unver N, McAllister F. IL-6 family cytokines: key inflammatory mediators as biomarkers and potential therapeutic targets. Cytokine Growth Factor Rev 2018; 41: 10–7. doi: 10.1016/j.cytogfr.2018.04.004 29699936 PMC6085880

[57] Zhang Y, Yang X, Bian F, Wu P, Xing S, Xu G, et al. TNF-α promotes early atherosclerosis by increasing transcytosis of LDL across endothelial cells: crosstalk between NF-κB and PPAR-γ. J Mol Cell Cardiol 2014; 72: 85–94. doi: 10.1016/j.yjmcc.2014.02.012 24594319

[58] Tong W, Chen X, Song X, Chen Y, Jia R, Zou Y, et al. Resveratrol inhibits LPS‑induced inflammation through suppressing the signaling cascades of TLR4‑NF‑κB/MAPKs/IRF3. Exp Ther Med 2020; 19(3): 1824–34. doi: 10.3892/etm.2019.8396 32104238 PMC7027153

[59] Nguyen TQ, Duy Binh T, Pham TL, Nguyen YD, Thi Xuan Trang D, Nguyen TT, et al. Anti-inflammatory effects of Lasia spinosa leaf extract in lipopolysaccharide-induced RAW 264.7 macrophages. Int J Mol Sci 2020; 21(10): 3439. doi: 10.3390/ijms2110343 32414062 PMC7279483

[60] Chistiakov DA, Melnichenko AA, Myasoedova VA, Grechko AV, Orekhov AN. Mechanisms of foam cell formation in atherosclerosis. J Mol Med (Berl) 2017; 95(11): 1153–65. doi: 10.1007/s00109-017-1575-8 28785870

[61] Chyu KY, Shah PK. HDL/ApoA-1 infusion and ApoA-1 gene therapy in atherosclerosis. Front Pharmacol 2015; 6: 187. doi: 10.3389/fphar.2015.00187 26388776 PMC4555973

[62] Calkin AC, Tontonoz P. Liver x receptor signaling pathways and atherosclerosis. Arterioscler Thromb Vasc Biol 2010; 30(8): 1513–8. doi: 10.1161/ATVBAHA.109.191197 20631351 PMC2919217

[63] Wang B, Tontonoz P. Liver X receptors in lipid signalling and membrane homeostasis. Nat Rev Endocrinol 2018; 14(8): 452–63. doi: 10.1038/s41574-018-0037-x 29904174 PMC6433546

[64] Li S, Cao H, Shen D, Jia Q, Chen C, Xing SL. Quercetin protects against ox‑LDL‑induced injury via regulation of ABCAl, LXR‑α and PCSK9 in RAW264.7 macrophages. Mol Med Rep 2018; 18(1): 799–806. doi: 10.3892/mmr.2018.9048 29845234 PMC6059709

[65] Nyandwi JB, Ko YS, Jin H, Yun SP, Park SW, Kim HJ. Rosmarinic acid increases macrophage cholesterol efflux through regulation of ABCA1 and ABCG1 in different mechanisms. Int J Mol Sci 2021; 22(16): 8791. doi: 10.3390/ijms22168791 34445501 PMC8395905

[66] Li J, Xiong T, Wang T, Wang M, Wang C, Yang F, et al. Baicalein targets CD36 to prevent foam cell formation by suppressing the excessive uptake of oxLDL and accelerating ABCA1-mediated cholesterol efflux in oxLDL-induced THP-1 macrophages. J Funct Foods 2022; 97: 105253. doi: 10.1016/j.jff.2022.105253

[67] Kattoor AJ, Goel A, Mehta JL. LOX-1: regulation, signaling and its role in atherosclerosis. Antioxidants 2019; 8(7): 218. doi: 10.3390/antiox8070218 31336709 PMC6680802

[68] Pothineni NVK, Karathanasis SK, Ding Z, Arulandu A, Varughese KI, Mehta JL. LOX-1 in atherosclerosis and myocardial ischemia: biology, genetics, and modulation. J Am Coll Cardiol 2017; 69(22): 2759–68. doi: 10.1016/j.jacc.2017.04.010 28571642

[69] Xu S, Ogura S, Chen J, Little PJ, Moss J, Liu P. LOX-1 in atherosclerosis: biological functions and pharmacological modifiers. Cell Mol Life Sci 2013; 70: 2859–72. doi: 10.1007/s00018-012-1194-z 23124189 PMC4142049

[70] Ou HC, Song TY, Yeh YC, Huang CY, Yang SF, Chiu TH, et al. EGCG protects against oxidized LDL-induced endothelial dysfunction by inhibiting LOX-1-mediated signaling. J Appl Physiol (1985) 2010; 108(6): 1745–56. doi: 10.1152/japplphysiol.00879.2009 20203069

[71] Qin M, Wang L, Li F, Yang M, Song L, Tian F, et al. Oxidized LDL activated eosinophil polarize macrophage phenotype from M2 to M1 through activation of CD36 scavenger receptor. Atherosclerosis 2017; 263: 82–91. doi: 10.1016/j.atherosclerosis.2017.05.011 28623741 PMC5557672

[72] Jiang Y, Wang M, Huang K, Zhang Z, Shao N, Zhang Y, et al. Oxidized low-density lipoprotein induces secretion of interleukin-1β by macrophages via reactive oxygen species-dependent NLRP3 inflammasome activation. Biochem Biophys Res Commun 2012; 425(2): 121–6. doi: 10.1016/j.bbrc.2012.07.011 22796220

[73] Guy E, Kuchibhotla S, Silverstein R, Febbraio M. Continued inhibition of atherosclerotic lesion development in long term Western diet fed CD36o /apoEo mice. Atherosclerosis 2007; 192(1): 123–30. doi: 10.1016/j.atherosclerosis.2006.07.015 16919281

[74] Park YM. CD36, a scavenger receptor implicated in atherosclerosis. Exp Mol Med 2014; 46(6): e99. doi: 10.1038/emm.2014.38 24903227 PMC4081553

[75] Duan J, Xiang D, Luo H, Wang G, Ye Y, Yu C, et al. Tetramethylpyrazine suppresses lipid accumulation in macrophages via upregulation of the ATP-binding cassette transporters and downregulation of scavenger receptors. Oncol Rep 2017; 38(4): 2267–76. doi: 10.3892/or.2017.5881 28791414

[76] Lin HC, Lii CK, Chen HC, Lin AH, Yang YC, Chen HW. Andrographolide inhibits oxidized LDL-induced cholesterol accumulation and foam cell formation in macrophages. Am J Chin Med 2018; 46(01): 87–106. doi: 10.1142/S0192415X18500052 29298513

[77] Chen XL, Liang PL, Gong MJ, Xu Y, Zhang L, Qiu XH, et al. Polyphenolics from Syzygium brachythyrsum inhibits oxidized low-density lipoprotein-induced macrophage-derived doam cell formation and inflammation. Foods 2022; 11(21): 3543. doi: 10.3390/foods11213543 36360156 PMC9656637

